# Selective internal radiation therapy (SIRT) for hepatocellular carcinoma (HCC): informing clinical practice for multidisciplinary teams in England

**DOI:** 10.1136/flgastro-2022-102137

**Published:** 2022-06-30

**Authors:** Helen L Reeves, John Reicher, Georgia Priona, Derek M Manas, Peter Littler

**Affiliations:** 1 Newcastle University Translational and Clinical Research Institute, Newcastle University, Newcastle upon Tyne, Tyne and Wear, UK; 2 Liver Unit, Newcastle upon Tyne Hospitals NHS Trust, Newcastle upon Tyne, UK; 3 Radiology, Newcastle upon Tyne Hospitals NHS Foundation Trust, Newcastle upon Tyne, UK; 4 Surgery, Newcastle upon Tyne Hospitals NHS Foundation Trust, Newcastle upon Tyne, UK

**Keywords:** HEPATOCELLULAR CARCINOMA, AUDIT, RADIOTHERAPY, CHEMOTHERAPY

## Abstract

**Objective:**

Hepatocellular carcinoma (HCC) deaths are rising alarmingly. Many patients are unsuitable for available therapies. Poor response rates further hamper outcomes for those that are. Selective internal radiation therapy (SIRT) offers hope, although which patients benefit over standard approaches remains unclear.

**Design/method:**

As a quality/service improvement, we audited consecutive patients treated with SIRT (2015-2020) by the Newcastle upon Tyne Hospitals National Health Service Foundation Trust HCC multidisciplinary team. Indications, Barcelona clinic liver cancer (BCLC) stage, treatment response, subsequent therapies and survival at 30 September 2021 were assessed

**Results:**

Fifty-one patients received SIRT. Thirty-day mortality was zero. Three months partial response, stable disease and progressive disease on imaging were 50%, 22% and 28%, respectively. Overall median survival was 21 months. There were four subgroups: (1) BCLC-B: HCC>7 cm too large for transarterial chemoembolisation (TACE) alone (n=21); (2) BCLC-B: HCC progressed post TACE (n=7); (3) BCLC-C: HCC with any combination of large tumour burden, branch portal vein thrombosis, non-hepatitis C virus aetiology (n=16); (4) BCLC-C: sorafenib inappropriate (n=7). In group 1, 5/21 (23.8%) of patients were downstaged to resection, 33% received subsequent medical therapies and median survival was >40 months. In BCLC-B patients treated second line (group 2), median survival was 14.2 months. In BCLC–C, median survival was 20.2 months for group 3 and 4.2 months for group 4.

**Conclusion:**

SIRT outcomes for advanced HCC, often bridging patients with adverse predictive factors to subsequent surgery or medical therapies, were encouraging. A role after TACE or for BCLC-C patients requires further assessment.

**WHAT IS ALREADY KNOWN ON THIS TOPIC**

- Selective internal radiation therapy (SIRT) is an alternative arterial therapy for patients with hepatocellular carcinoma (HCC). Although randomised control trial evidence showing superiority over standard therapies is lacking, it is a well-tolerated treatment with excellent outcomes for some patients. The National Institute for Health and Care Excellence (NICE) has approved its use in National Health Service (NHS) Trusts in England within a multidisciplinary team (MDT) setting. Decision-making aids within MDTs are needed.

**WHAT THIS STUDY ADDS**

- On an individual funding request basis, SIRT has been available for highly selected patients with advanced HCC in the Newcastle NHS Foundation Trust and we have audited our practice over 4.5 years. Overall, 51 (6.8%) of new patients were treated with SIRT, which was well tolerated—even in older patients with comorbid conditions. In 48%, SIRT facilitated further treatments—including 10% downstaged to resection and longer-term survival.

**HOW THIS STUDY MIGHT AFFECT RESEARCH, PRACTICE OR POLICY**

- Following NICE guidance, NHS Trusts are expected to consider SIRT as an option for patients with HCC within an MDT setting. Highly experienced interventional teams throughout England already have the skills to offer this, although support and technical training on dosimetry and delivery of the options approved are needed. These data can be used by MDTs in parallel at the outset, to aid appropriate patient selection, with the expectation that future guidance will be informed by wider experience, audit and clinical trials.

## Introduction

Hepatocellular carcinoma (HCC) is the third leading cause of cancer death globally.^1^ For early-stage cancers curative treatments such as resection, liver transplantation and ablation are considered. For those with multifocal tumours and preserved liver function, transarterial chemoembolisation (TACE) has been the mainstay of treatment.^2^ Unfortunately, the majority of patients with HCC in England present with advanced stage disease.^3 4^ Advanced HCC includes patients with greater size and numbers of tumours, portal vein invasion, metastatic spread, as well patients with deteriorating liver function and performance status regardless of tumour burden. HCC typically complicates underlying chronic liver disease and it is often liver function or comorbid conditions that limit treatment options. The importance of combination staging is widely recognised, with the Barcelona clinic liver cancer (BCLC) guideline^2^ and modifications of it,^5^ commonly used to aid treatment selection. Over the last decade, medical treatments for advanced disease have been introduced—with multikinase inhibitors available in both first-line (sorafenib, lenvatinib) and second-line (regorafenib) settings. The combination of atezolizumab immunotherapy and the vascular endothelial growth factor inhibitor bevacizumab (atezo/bev) is now also approved first line.^6^ In practice though, not all patients respond to medical therapies and few are ‘downstaged’ to surgical intervention. Additional therapeutic options are needed.

Radioembolisation, or selective internal radiation therapy (SIRT), is an alternative arterial therapy—delivering the cytotoxic radioisotope Yttrium-90 to cancers, rather than the typical chemoembolic beads used in TACE. SIRT has shown promise—with the potential to treat larger tumours. For TACE, benefit is reduced in tumours greater than 7 cm.^7^ Early studies also suggested SIRT benefit for patients with branch portal vein thrombosis (PVT)—PVT being an independent poor prognostic factor and one which is a contraindication to treatment with chemoembolisation.

There has not been a large randomised control trial (RCT) comparing the efficacy of SIRT with TACE, but two RCTs have compared the efficacy of SIRT to sorafenib. SARAH (sorafenib versus radioembolization in advanced HCC)^8^ was a French trial that included patients with varied underlying aetiologies of liver disease, with either Child-Pugh A or B liver function. Although SIRT was well tolerated, there were no differences in progression-free or overall survival between the sorafenib and SIRT treated groups. The SIRveNIB (selective internal radiation therapy versus sorafenib) trial^9^ was based in the Asia Pacific region. Similarly, there were fewer adverse events reported with SIRT, but no differences in survival. As these trials failed to meet their primary endpoints of showing survival superiority, there is no RCT evidence base on which to recommend treatment with SIRT. Personalised dosimetry, with the delivery of tumour doses above 100 Gy with resin^10^ or 205 Gy with glass microspheres^11^ to advanced tumours, may achieve greater responses and improved overall survival compared with standard generic SIRT dosing. However, SIRT is not currently recommended for the treatment of patients with BCLC-C stage HCC.^6^

In parallel, physicians, surgeons and healthcare providers have recognised that there are some patients where SIRT can play an important role, despite the lack of RCT evidence. In large retrospective series, improved survival in responders has been reported,^12^ with the LEGACY (local radioembolization using glass microspheres for the assessment of tumor control with Y-90) study published in 2021.^13^ LEGACY was a multicentre retrospective single arm study, in which 162 consecutive patients with solitary HCC≤8 cm, median tumour size 2.6 cm, Child-Pugh A cirrhosis and Eastern Cooperative Oncology Group performance status 0–1, were treated with radioembolisation. Clinically meaningful outcomes were observed, with prolonged durations of response and subsequent transplantation and resection subsequently performed in 21% and 6.8%, respectively.^13^ Consequently, the option to consider radioembolisation for BCLC-0 to BCLC-B stage patients has been recognised within the BCLC guideline.^13^

In 2020–2021, the role of SIRT was reviewed in England by the National Institute for Health and Care Excellence (NICE), acting as an advisor to the National Health Service (NHS). Recognising that SIRT may have advantages for some patients acknowledging that many UK patients (older, lacking cirrhosis, with metabolic syndrome associated comorbidities)^3^ were not well represented in the evidence base underpinning international guidelines, NICE supported SIRT within a multidisciplinary team (MDT) setting as an option for treating unresectable advanced HCC in patients with Child-Pugh grade A liver impairment when conventional transarterial therapies are inappropriate.^14^ This is in keeping with the wider realisation that while evidence-based guidelines are immensely helpful, patient-specific characteristics and a centres expertise are important considerations when implementing a more personalised approach, with a positive impact on patient outcomes.^15^ The decision by NICE has been welcomed by the healthcare providers, patients and their advocacy groups. However, the absence of ‘a guideline’ presents a challenge, especially in centres where SIRT has not been accessible and expertise is currently lacking.

Supported by the Newcastle upon Tyne NHS Foundation Trust (NUTH), led within our hepatopancreatobiliary (HPB) MDT, treatment with SIRT has been available for 7 years. We have audited our MDT practice, presented here with the aim of aiding decision-making for ‘real-world’ patients in England.

## Materials and methods

In NUTH, between January 2015 and June 2020, SIRT treatment was available on an individual-named patient basis. HPB MDT review prior to its use was essential, with patient outcomes subject to audit as part of a quality/service improvement project (NUTH 10826). Glass microspheres were provided by Boston Scientific UK (2015–2019) and BTG thereafter. Selected patients were those where standard first-line therapies were not ideal. All patients had advanced disease, with Child-Pugh Grade A liver function. The majority were unsuitable for resection without prior downstaging, or had features associated with poorer responses to first-line TACE—based on a combination of lesion size, distribution or presence of PVT. This included patients with single lesions>7 cm^7^ and those with partial (segmental or lobar branch) PVT. As features associated with poorer responses to sorafenib, large size and non-HCV-associated HCC aetiology^16^ were also considered in elderly patients with non-cancer-associated comorbidities impacting their quality of life. A pre-SIRT procedure was performed 2 weeks prior to SIRT, comprising angiography, cone-beam CT and injection of 150MBq Tc-99 macroaggregated albumin at the intended microsphere injection position(s). Treatment was administered as an in-patient, with clinical nurse specialist support. Data were collected retrospectively from the electronic patient record and Picture Archiving and Communication System, including indications for SIRT, disease response rates calculated according to modified response evaluation criteria in solid tumours (mRECIST)^17 18^ and survival with a minimum of 1-year follow-up at 30 September 2021. Data analyses were performed using IBM SPSS Statistics 25 licensed to Newcastle University.

## Results

### Patient characteristics

A total of 51 patients with HCC were treated, with characteristics summarised in table 1. The median age was 72 years (range 39–84). Just over 80% were men. Fatty liver disease (one-third attributed to alcohol excess; two-thirds to obesity and the metabolic syndrome) was the most common underlying aetiology. Overall, 8/51 (15.7%) had no recognised chronic liver disease, while a further 12 (23.5%) had chronic liver without established cirrhosis. The majority had a European Co-operative Oncology Group (ECOG) performance status of 0–1, with 8/51 graded as 2. Overall, 39 (76.5%) had unilobar disease with 30 (58.8%) having a single lesion. The size of the largest lesion ranged from 3.3 to 19.1 cm (median 8.5 cm). Twelve patients (23.1%) had branch portal vein involvement. None had main portal vein involvement. In two-thirds, SIRT was administered first line. The median total activity of Yttrium-90 delivered was 3.2GBq.

**Table 1:** Patient characteristics and outcomes, considering all patients and 4 subgroups as recognised by the HPB MDT

	All patients	Group 1 BCLC-B too large for TACE alone	Group 2 BCLC-B progression post TACE	Group 3 BCLC-C sorafenib eligible	Group 4 BCLC-C sorafenib unsuitable
Patient number	51	21	7	16	7
age—median (range)	72 (39–84)	72 (51–84)	76 (62–81)	68 (50–81)	72 (39–84)
Sex M/F	41/10	15/6	6/1	16/0	4/3
Aetiology—no CLD	8	3	0	2	3
ARLD	9	3	3	3	0
NAFLD	19	10	2	5	2
HCV	9	3	0	5	1
Other	6	2	2	1	1
Cirrhosis N/Y	20/31	11/10	1/6	5/11	3/4
Child-Pugh A	51	21	7	16	7
ECOG PST 0/1/2	21/22/8	14/7/0	3/4/0	4/8/4	0/3/4
Tumour number 1/>1	30/21	14/7	2/5	10/6	4/3
Size—median, cm (range)	8.5 (3.3–19.1)	9.7 (5.3–19.1)	5.9 (3.3–11.0)	8.8 (3.7–13.0)	8.3 (3.8–17.0)
Total activity—median GBq (range)	3.2 (1.04–10.77)	3.93 (1.49–10.77)	2.32 (1.04–3.20)	3.20 (1.61–6.67)	2.8 (1.26–6.53)
Branch PVT N/Y	39/12	21/0	7/0	6/10	5/2
Unilobar Y/N	39/12	16/5	5/2	13/3	5/2
Prior therapy Y/N	17/34	2/19	7/0	5/11	3/4
Next therapies					
Resection	5	5	0	0	0
Active monitoring	3	3	0	0	0
Further SIRT	1	0	0	1	0
Medical 1 L	8	1	2	5	0
Medical 1l+2 L	7	3	0	3	1
Supportive care	27	9	5	7	6
mRECIST PR/SD/PD (3 months)	25/11/14	12/6/2	2/0/5	7/5/4	4/0/3
	**Median survival (months)**				
All—median	20.17	Ongoing	14.8	20.2	4.2
Resection n=5	Ongoing	All alive	–	–	–
Non-resected	15.5	21.7	–	–	–
First-line SIRT	21.67	Ongoing	–	27.4	3.7
Second-line SIRT	10.93	42.1	14.8	10.7	7.3
Unilobar	19.87	Ongoing	11.4	27.4	4.2
Bilobar	10.93	20.2	14.8	8.8	3.2
+PVT	10.7	–	–	10.7	2.7

ARLD, alcohol related liver disease; BCLC, Barcelona clinic liver cancer; CLD, chronic liver disease; ECOG PST, European Co-operative Oncology Group Performance Status; F, female; GBq, gigabecquerel; HCV, hepatitis C virus; 1L, first line; 2L, second line; M, male; mRECIST, modified response evaluation criteria in solid tumours; N, no; NAFLD, non-alcoholic fatty liver disease; PVT, portal vein thrombus; SIRT, selective internal radiation therapy; TACE, transarterial chemoembolisation; Y, yes.

### Patient outcome

The treatment was well tolerated and 30-day mortality was 0. Overall, 50/51 patients had evaluable disease on imaging at 3 months, with 36/50 (72%) achieving at least a partial response or stable disease (table 1). At the time of last follow-up, five (9.8%) patients had gone on to have liver resection and remained alive. Two developed recurrence—one at 47.5 and one at 63.3 months post SIRT. Both are undergoing further treatment. Three (5.9%) remain under active follow-up without progression (median 26.1 months). Of the total 51 patients, 43 (82.4%) have progressed. One received further SIRT, with 15/43 (34.9%) having received subsequent medical therapies. Overall, 27/51 (52.9%) received supportive care second line. The median survival of the entire cohort was 20.17 months.

### Subgroup analysis

Patients characteristics and outcomes were analysed within the categories for which SIRT treatment was advised within the MDT. The categories are detailed in table 1, including group (1) BCLC-B with HCC>7 cm (n=21); group (2) BCLC-B with HCC progression post TACE (n=7); group (3) BCLC-C with HCC feature associated with lesser response to sorafenib (large tumour burden, PVT, n=16); group (4) BCLC-C with a reason to avoid sorafenib (n=7).

**Figure 1:**
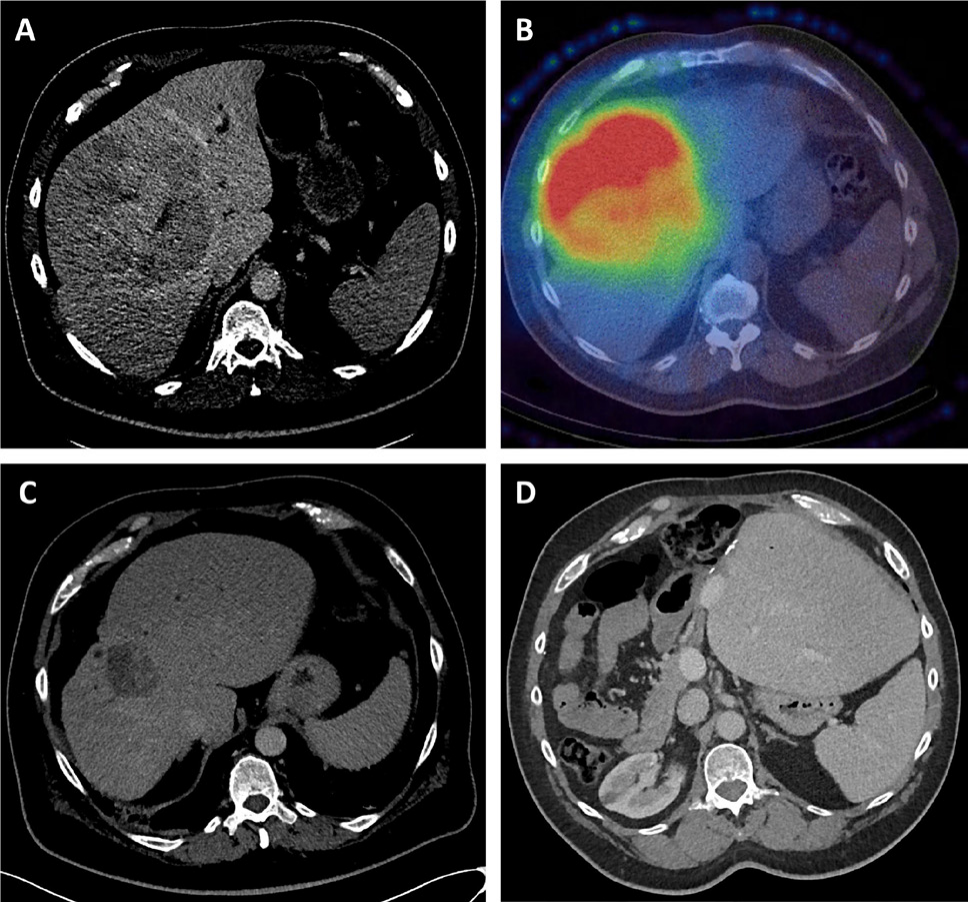
A selective internal radiation therapy (SIRT) case study. A 56-year-old non-cirrhotic patient had a 14 cm moderately differentiated hepatocellular carcinoma in the right liver lobe and segment 4, with a small left lateral lobe (future liver reminant (FLR)) (A). Single-photon emission computed tomography (CTSPECT) post SIRT demonstrated high uptake of SIRT within the tumour (B). CT scan at 18 months, following a second SIRT delivering 350 Gy to the tumour, demonstrated a reduction in tumour volume (from 1695 cc to 63 cc) with hypertrophy of the FLR (C), following which a curative resection was performed. CT scan 5 years later confirmed the patient remains free of tumour recurrence.

BCLC-B patients in group 1 receiving first-line SIRT did well. Two received selective TACE in addition, as part of their initial treatment, to smaller distinct HCC outwith the SIRT-targeted lobes(s). The majority had further treatment, including 25% downstaged to resection (median size 15 cm), 14.3% remaining under active monitoring with stable disease and 19% receiving medical therapies after progression. Twelve are alive with a median survival in excess of 40 months. A case downstaged to resection is summarised in figure 1.

BCLC-B patients in group 2 were those treated with SIRT having developed recurrence or progression post first-line TACE (median TACE treatments 2 (range 1–7)). Two were subsequently treated with medical therapy (sorafenib), with an overall median survival of 14.8 months.

BCLC–C patients in group 3 were those considered for either sorafenib or SIRT in our MDT, with the options discussed with the patients. Typically, these were patients with comorbidities, large volume unilobar disease or portal vein invasion. In the first-line setting, the median survival of these patients was 27.4 months, with a median survival of 10.7 months for those with PVT.

BCLC-C patients in group 4 were a small eclectic group with advanced HCC and extenuating circumstances, offered SIRT after MDT discussion. These included patients with significant immunosuppression, inflammatory disease with impaired mobility and those intolerant of sorafenib. Median survival in this group was 4.2 months.

## Discussion

Although there is uncertainty about outcomes guided by RCT, NICE guidance approves NHS funding for SIRT treatment in unresectable patients with HCC and Child-Pugh A liver function, who are not suitable for conventional TACE. The guidance advises that SIRT beyond these criteria may be considered subject to a local MDT decision and funding availability within individual NHS Trusts.

Here, we have reviewed current evidence and described the practice of our MDT over a 4.5-year period, when SIRT was available to selected patients on a named patient basis, subject to review after a clinical care quality audit. We now incorporate this experience into our MDT decision-making, while noting the limitations—being from a single centre without a comparable control group of patients synchronously receiving standard care. Furthermore, the standard of care options for patients with advanced BCLC-C HCC, or with BCLC-B progressive HCC post TACE, have changed in the last 5 years—with the approval of additional medical therapies.

The first noteworthy consideration, is that the number of patients treated with SIRT over the 4.5-year period in our centre, was relatively small, accounting for 51 (6.8%) of ~750 new referrals to our MDT in the same period. This reflects the use of SIRT in preference to standard therapies, in relatively small numbers, where our MDT considered it the best personalised treatment. The approval of SIRT by NICE is broadly in line with this approach.

Within the treated patient groups, the data from our audit support that of the DOSISPHERE-01 study^11^ and the role of SIRT for BCLC-B patients with HCC>7 cm, especially for unilobar or single lesions. In this group of patients, further therapies were facilitated in over 50%, with downstaging to surgical resection in 25%. As our patients had intermediate to advanced stage HCC, downstaging to liver transplantation was not addressed. However, the LEGACY study supports the use of SIRT in patients with single lesions of any size<8 cm in this setting.^13^ Presently, there is insufficient evidence that for smaller lesions, SIRT is superior to TACE and NICE guidance points to the use of TACE first line where possible. SIRT selection over TACE would need justification in an MDT setting. MDTs might focus on those less likely to achieve effective bridging responses with combinations of TACE and ablation (eg, multifocal disease; single lesion approaching 5 cm; those at risk of decompensation post bridging therapy). Ideally, a change in practice of this nature would be subject to national co-operation and audit, informing future practice.

The outcomes for our BCLC-B patients with progressive disease post TACE were encouraging, but atezo/bev medical therapy is now an option for these patients.^19^ SIRT should be reserved for those in whom atezo/bev is not advisable (eg, immune disease; uncontrolled portal hypertension), with decision-making balanced against availability and suitability for other medical therapies and clinical trials. The outcomes of trials assessing the role of SIRT in combination with immunotherapies (eg, DOORwaY90, NCT04736121; NASIR-HCC, NCT03380130; MEDI4736, NCT04522544) are awaited and may inform changes in future practice, with earlier use of SIRT.

The outcomes for BCLC-C group 3 patients with unilobar HCC, as well as those with portal vein invasion, were also encouraging. Again though, large tumour burden and portal vein invasion are not features associated with resistance to atezo/bev. For patients with a single large lesion, a SIRT discussion in an MDT setting is reasonable, recognising the benefits of a single well-tolerated treatment that does not preclude future medical therapies.

The outcome for the heterogenous BCLC-C group 4 patients was not as good. Having said that, these were patients with advanced stage, where supportive care was the only alternative. Their poorer outcome as a group was not unexpected, with some individuals deriving significant benefit. In line with NICE guidance, within an MDT setting and pending approval of funding, SIRT may be considered for these patients.

### Overview and recommendation

**Figure 2:**
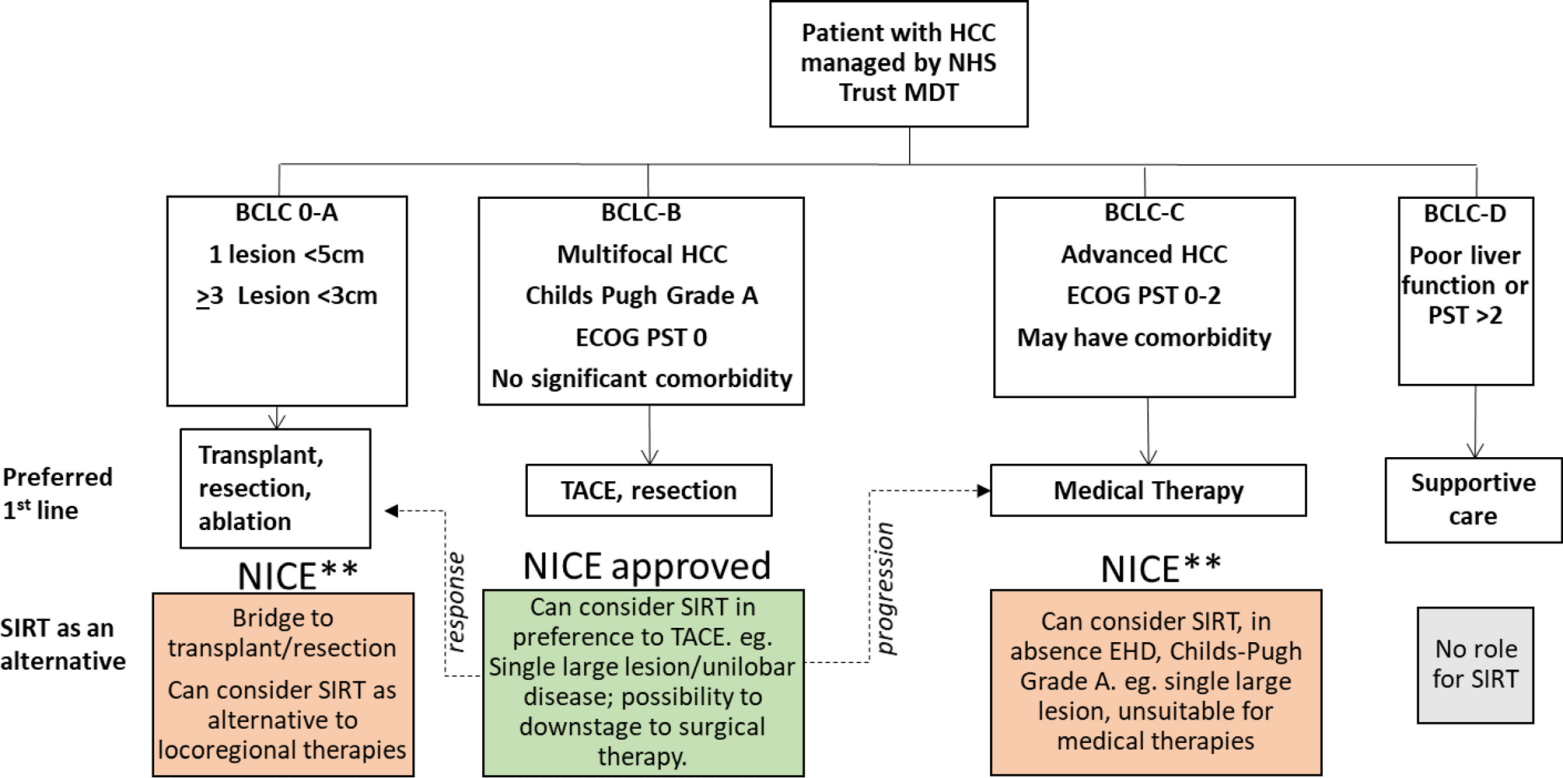
Summary of recommendation for selective internal radiation therapy (SIRT) use within an England multidisciplinary team (MDT) setting. In England, patients with hepatocellular carcinoma (HCC) are managed within the National Health Service (NHS), referred to specialist centre MDTs in tertiary referral centre hospital trusts. The MDTs stage the patient, with the Barcelona clinic for liver cancer (BCLC) algorithm commonly used as a guide-aiding treatment selection by the MDT. The preferred first-line therapies for patients within each stage are shown. SIRT has been approved by the National Institute for Health and Care Excellence (NICE), as an alternative to transarterial chemoembolisation (TACE) first line, typically used for BCLC-B patients, if an MDT considers SIRT a more suitable option (green box). For BCLC-B patients responding to or downstaged by SIRT, subsequent treatments for earlier stage disease may be considered (dotted line to left). For BCLC-B patients who progress post SIRT, medical therapies would be considered (dotted line to right). NICE advised that SIRT for patients with BCLC 0-A, or BCLC-C stage HCC (highlighted ** and shown in orange boxes) could be considered as an alternative to preferred first-line therapies within the setting of an expert MDT, but offered subject to funding approval. ECOG PST, European Co-operative Oncology Group Performance Status; EHD, extrahepatic disease.

We all recognise that we should offer evidence-based therapies wherever possible, as well as support ongoing RCTs to inform future guidelines. However, regional access to services and RCTs is not equitable in the UK. Furthermore, many patients with HCC (older, with comorbidities) are either unsuitable for RCT inclusion or prefer not to travel to take part in RCTs. Regional MDTs play to their strengths, developing personalised approaches to suit the patients served. Within our NUTH MDT, we have explored SIRT and we are now in a position to offer it. Our recommendations are summarised in figure 2, advising limited deviation from evidence-based practice, but supporting local MDTs discretion when discussing available personalised approaches with their patients. With careful patient selection, the cost implications for individual NHS Trusts and Integrated Care Systems should be acceptable, while still ensuring that the necessary skills and expertise for SIRT delivery are developed and maintained. While adopting this tactic, it is essential that all MDTs keep abreast of the rapidly changing landscape of treatment opportunities for patients with advanced HCC, moving to incorporate these as the evidence base evolves.

## Data Availability

Data are available upon reasonable request. The summary of the audit dataset is included in the manuscript. No individual identifiable patient information is included. To protect their identities—given the small numbers of patients, receiving a specific treatment in a defined time frame in an identified NHS Trust, the actual dataset is not provided.
